# Comparative Diagnostic Performance of a Multimodal Large Language Model Versus a Dedicated Electrocardiogram AI in Detecting Myocardial Infarction From Electrocardiogram Images: Comparative Study

**DOI:** 10.2196/75910

**Published:** 2025-09-17

**Authors:** Haemin Lee, Sooyoung Yoo, Joonghee Kim, Youngjin Cho, Dongbum Suh, Keehyuck Lee

**Affiliations:** 1 Department of Emergency Medicine, Seoul National University Bundang Hospital, Seongnam-si, Gyeonggi-do, Republic of Korea; 2 ARPI Inc, Seongnam-si, Gyeonggi-do, Republic of Korea; 3 Office of eHealth Research and Businesses, Seoul National University Bundang Hospital, Seongnam-si, Gyeonggi-do, Republic of Korea; 4 Department of Internal Medicine, Cardiovascular Center, Seoul National University Bundang Hospital, Seongnam-si, Gyeonggi-do, Republic of Korea; 5 Department of Family Medicine, Seoul National University Bundang Hospital, 82 Gumi-ro 173 beon-gil, Bundang-gu, Seongnam-si, Gyeonggi-do, 13620, Republic of Korea, 82 10-2514-7428

**Keywords:** artificial intelligence, myocardial infarction, ECG, LLMs, large language models, electrocardiogram

## Abstract

**Background:**

Accurate and timely electrocardiogram (ECG) interpretation is critical for diagnosing myocardial infarction (MI) in emergency settings. Recent advances in multimodal large language models (LLMs), such as ChatGPT (OpenAI) and Gemini (Google DeepMind), have shown promise in clinical interpretation for medical imaging. However, whether these models analyze waveform patterns or simply rely on text cues remains unclear, underscoring the need for direct comparisons with dedicated ECG artificial intelligence (AI) tools.

**Objective:**

This study aimed to evaluate the diagnostic performance of ChatGPT and Gemini, a general-purpose LLM, in detecting MI from ECG images and to compare its performance with that of ECG Buddy (ARPI Inc), a dedicated AI-driven ECG analysis tool.

**Methods:**

This retrospective study evaluated and compared AI models for classifying MI using a publicly available 12-lead ECG dataset from Pakistan, categorizing cases into MI-positive (239 images) and MI-negative (689 images). ChatGPT (GPT-4o, version November 20, 2024) and Gemini (Gemini 2.5 pro) were queried with 5 MI confidence options, whereas ECG Buddy for Microsoft Windows analyzed the images based on ST-elevation MI, acute coronary syndrome, and myocardial injury biomarkers.

**Results:**

Among 928 ECG recordings (239/928, 25.8% MI-positive), ChatGPT achieved an accuracy of 65.95% (95% CI 62.80‐69.00), area under the curve (AUC) of 57.34% (95% CI 53.44‐61.24), sensitivity of 36.40% (95% CI 30.30‐42.85), and specificity of 76.2% (95% CI 72.84‐79.33). With Gemini 2.5 Pro, accuracy dropped to 29.63% (95% CI 26.71‐32.69), AUC to 51.63% (95% CI 50.22‐53.04), and sensitivity rose to 97.07% (95% CI 94.06‐98.81), but specificity fell sharply to 6.24% (95% CI 4.55‐8.31). However, ECG Buddy reached an accuracy of 96.98% (95% CI 95.67‐97.99), AUC of 98.8% (95% CI 98.3‐99.43), sensitivity of 96.65% (95% CI 93.51‐98.54), and specificity of 97.10% (95% CI 95.55‐98.22). DeLong test confirmed that ECG Buddy significantly outperformed ChatGPT (all *P*<.001). In a qualitative error analysis of LLMs’ diagnostic explanations, GPT-4o produced fully accurate explanations in only 5% of cases (2/40), was partially accurate in 38% (15/40), and completely inaccurate in 58% (23/40). By contrast, Gemini 2.5 Pro yielded fully accurate explanations in 32% of cases (12/37), was partially accurate in 14% (5/37), and completely inaccurate in 54% (20/37).

**Conclusions:**

LLMs, such as ChatGPT and Gemini, underperform relative to specialized tools such as ECG Buddy in ECG image–based MI diagnosis. Further training may improve LLMs; however, domain-specific AI remains essential for clinical accuracy. The high performance of ECG Buddy underscores the importance of specialized models for achieving reliable and robust diagnostic outcomes.

## Introduction

Electrocardiogram (ECG) interpretation is a fundamental skill in cardiovascular medicine, playing a crucial role in diagnosing conditions such as ST-elevation myocardial infarction (STEMI), arrhythmias, and electrolyte imbalances [1]. Accurate and timely ECG analysis is critical in clinical decision-making, particularly in emergency settings where rapid interventions impact patient outcomes.

With advancements in artificial intelligence (AI), researchers have explored using various deep learning techniques, including convolutional neural networks and transformer-based models, to automate ECG interpretation by extracting clinically relevant features from ECG signal or image data [2-5].

Recently, multimodal large language models (LLMs) trained on textual and imaging data have gained attention in the medical field [6]. These models have demonstrated the ability to generate diagnostic reports, highlighting their potential for medical image interpretation [7]. As LLMs have become increasingly sophisticated, the interest in applying similar multimodal architectures to ECG interpretation has also grown.

General-purpose LLMs, such as ChatGPT (OpenAI), have recently demonstrated some capabilities in assisting with image interpretation and text-based medical assessments [8,9]. Unlike traditional AI models specifically trained for ECG signal processing, these models leverage extensive general knowledge and are now being considered for processing visual medical data, including ECG images. For example, Zaboli et al [10] investigated the ECG interpretation ability and outcome prediction of ChatGPT in the emergency department and found moderate agreement with cardiologists, but with notable discrepancies in major adverse cardiac event risk assessment. Zhu et al [11] reported that GPT-4 achieved approximately 83% accuracy in multiple-choice ECG diagnostic questions. Günay et al [12] compared GPT-4, GPT-4o, and Gemini Advanced (Google DeepMind) against cardiologists and emergency medicine specialists using routine and challenging ECG cases. Although all LLMs underperformed compared to cardiologists, GPT-4o showed relatively better accuracy and moderate agreement, suggesting potential as a supportive tool in clinical settings. Similarly, Avidan et al [13] examined the ability of GPT-4o to detect atrial fibrillation in ECGs with confounding factors. Their findings indicated that while the overall accuracy of GPT-4o was comparable to that of internists and primary care physicians, it fell short of cardiologists’ performance, particularly in challenging scenarios. In contrast, Günay et al [14] reported that GPT-4 outperformed emergency medicine specialists in interpreting everyday ECG cases and performed on par with cardiologists when facing more complex ECG challenges. However, a key limitation of their study is that ECG descriptions rather than actual ECG images were evaluated by GPT-4, potentially limiting its applicability in clinical settings.

Collectively, these studies highlight that although LLM-based approaches in ECG interpretation hold promise, their reliability in complex cases remains limited. Moreover, it remains unclear whether these models truly analyze waveform patterns or simply rely on text-based cues, such as machine-readable annotations. This raises concerns about the reproducibility of the models’ interpretations when presented with raw ECG images alone.

To date, no study has systematically compared the performance of LLMs against specialized ECG diagnostic AI tools. This comparison is becoming increasingly relevant, as general-purpose LLMs are not specifically designed for cardiovascular medicine. However, speculation about their potential applications in ECG interpretation is already widespread. Thus, a comparative evaluation with dedicated ECG AI software is necessary to determine the feasibility of LLM-based ECG interpretation in clinical practice.

Recent studies comparing ChatGPT-4o with Gemini on ECG interpretation tasks identify both models as suitable reference LLMs, so we included them in our evaluation [12]. We then benchmarked their performance against ECG Buddy (ARPI Inc.), a commercially available, domain-specific AI tool for ECG analysis.

ECG Buddy is approved by the Korean Ministry of Food and Drug Safety, South Korea’s regulatory agency for medical device oversight, responsible for thorough examinations and continuous supervision, and is currently in routine clinical use at multiple hospitals, including tertiary care centers. ECG Buddy has been validated in multiple studies, demonstrating superior diagnostic accuracy to clinical experts in detecting conditions such as myocardial infarction (MI), hyperkalemia, and right ventricular (RV) dysfunction [15-18].

This study aimed to evaluate the diagnostic performance of ChatGPT and Gemini relative to a dedicated ECG AI (ECG Buddy) in analyzing ECG images for MI detection. MI interpretation is one of the most essential aspects of ECG analysis. Through this comparative study, we aimed to determine whether LLMs could currently be used for ECG interpretation in clinical practice.

## Methods

### Study Design and Data Preparation

In this retrospective study, we evaluated the performance of ChatGPT, Gemini, and ECG Buddy in classifying MI from ECG images. A publicly available 12-lead ECG image dataset compiled by the Ch. Pervaiz Elahi Institute of Cardiology in Multan, Pakistan, was used [19]. The dataset includes ECG images categorized into the following 4 groups: patients with MI (239 images), patients with abnormal heartbeats (233 images), patients with a history of MI (172 images), and healthy controls (284 images). This publicly available, fully deidentified ECG image dataset was chosen to enable reproducible benchmarking without privacy constraints and is frequently referenced in prior studies. The dataset does not provide additional patient information beyond these labels. It lacks metadata such as infarct territory or cardiac biomarker data needed to differentiate STEMI from non-STEMI (NSTEMI). Therefore, further analyses by infarct location or NSTEMI status were not possible.

This study was designed and reported in accordance with the TRIPOD-LLM (Transparent Reporting of a Multivariable Prediction Model for Individual Prognosis Or Diagnosis specifically tailored for LLM) guidelines, a comprehensive reporting framework for studies involving LLMs in health care, to ensure that every step, from data processing and image-to-text conversion to AI querying and performance evaluation, was transparently and reproducibly documented [20]. To ensure consistency in data processing, extraneous areas of the images, including any supplementary text not related to patient information or diagnosis, were cropped, retaining only the waveform regions. No raster-to-signal conversion was applied, and all analyses were performed directly on the image data. For classification, only the images labeled as “patients with myocardial infarction” were designated as MI-positive, representing active MI cases. The remaining 689 images, comprising abnormal heartbeats, history of MI, and healthy cases, were classified as MI-negative.

### AI Query and Output (ChatGPT and Gemini)

The identical workflow was applied to 2 multimodal LLMs—GPT-4o (OpenAI, version November 20, 2024) and Gemini 2.5 Pro (Google, May 2025 release). To assess the ability of LLMs to classify MI from ECG images, we designed a structured prompt aimed at systematically capturing the model’s diagnostic rationale and confidence in detecting MI. Before querying, ECG images were converted into base64 format, a lossless binary-to-text format required by both application programming interfaces (APIs) and one that preserves every pixel and ensures no loss of ECG signal fidelity.

Each LLM received the base64-encoded images and prompted explicitly to analyze them, determine the likelihood of MI, and select from 5 predefined response categories—unknown, unlikely, possible, probable, and definite—representing increasing diagnostic certainty. Both LLMs were queried with the prompt shown in Textbox 1, which presents the ChatGPT-4o version; the Gemini 2.5 Pro prompt was identical, differing only in that the model name was replaced with “GPT-4o.”

**Textbox 1. GPT-4o prompt design.**

SYSTEM_TEXT = (

“Analyze the provided ECG image in base64 format and assess the likelihood of Myocardial Infarction (MI).”

“Based on your analysis, select the most appropriate confidence level regarding the presence of MI and provide a detailed explanation for your choice.”

“Specify which leads exhibit abnormalities and describe the observed changes (eg, ST-segment elevation, T-wave inversion, pathological Q waves).”

“If no abnormalities are present, explain why the ECG appears normal. If the findings are ambiguous, discuss potential differential diagnoses.”

)

CONFIDENCE_TEXT = (

“Confidence Levels for MI Diagnosis:\n”

“Definitely Not – No ECG evidence or extremely low probability of MI; clearly normal waveform patterns.\n”

“Unlikely – Minimal or questionable evidence making MI improbable; non-specific changes.\n”

“Possible – Moderate suspicion with mixed findings; abnormalities with alternative explanations possible.\n”

“Probable – Strongly suggestive findings (ST-segment elevation, pathological Q waves, T-wave inversion).\n”

“Definite – Conclusive evidence: clear ST elevation in contiguous leads, significant Q waves, reciprocal changes.\n\n”

“Respond in JSON with keys: confidence, explanation, abnormal_leads.”

)

data = [] for idx, (file_name, base64_image) in enumerate(tqdm(image_results, desc=“ECG”)):

try:

messages = [

{“role”: “system”, “content”: SYSTEM_TEXT},

{

“role”: “user”,

“content”: [

{

“type”: “image_url”,

“image_url”: {

“url”: f"data:image/png;base64,{base64_image}",

“detail”: “high”

},

},

{

“type”: “text”,

“text”: CONFIDENCE_TEXT,

},

],

},

]

response =client.chat.completions.create(

model=“gpt-4o”,

messages =messages,

temperature =0.0,

max_tokens =300,

)

chatgpt_result =response.choices[0].message.content.strip()

except Exception as e:

print(f”[Error] {file_name}: {e}")

chatgpt_result = None

data.append({“File Name”: file_name, “ChatGPT Result”: chatgpt_result})

**Figure 1.**
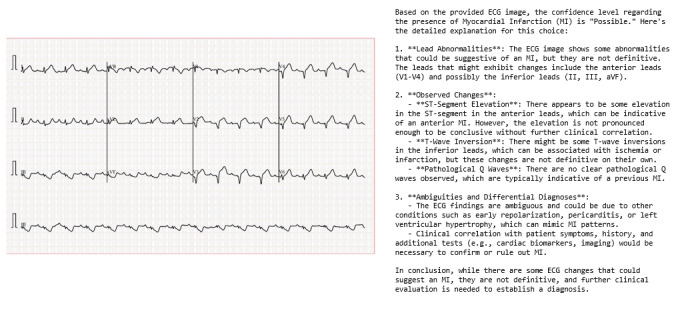
Example electrocardiogram images and ChatGPT model output for myocardial infarction detection. ECG: electrocardiogram.

To define a positive MI diagnosis based on this likelihood measure, the Youden index was applied to determine an optimal cutoff. Specifically, ChatGPT was instructed to identify and specify which ECG leads exhibited abnormalities, describe the changes observed (such as ST-segment elevation, T-wave inversion, or pathological Q waves), and provide detailed reasoning supporting its diagnostic conclusion. In cases where no abnormalities were noted or the ECG findings were ambiguous, the model was prompted to discuss potential alternative diagnoses or clearly explain why the ECG appeared normal. All queries were conducted using GPT-4o via the ChatGPT API. An example of a typical ChatGPT response is illustrated in Figure 1. All inferences were conducted as single-turn prompts, since each case consisted only of a deidentified ECG image with no ancillary clinical or serial ECG data that could support further interaction, and our study aimed to evaluate the models’ final diagnostic performance.

### Qualitative Assessment of Diagnostic Explanations–ChatGPT and Gemini

In addition, a qualitative assessment of the diagnostic explanations of LLMs was performed to further evaluate the ability to accurately interpret ECG images. For GPT-4o, 40 cases were randomly selected: 10 true-positive, 10 true-negative, 10 false-positive, and 10 false-negative. For Gemini 2.5 Pro, the same procedure yielded 37 cases (10 true-positive, 10 true-negative, 10 false-positive, and 7 false-negative) owing to the model’s smaller false-negative pool. Two board-certified clinicians, an emergency medicine specialist and a cardiologist, each with more than 10 years of ECG interpretation experience, independently reviewed the diagnostic explanations to assess whether the model provided clinically appropriate rationales for their classification. They then reconciled any discrepancies by consensus, and the final consensus ratings, together with per-reviewer tallies, are reported in the Results section.

### AI-Powered Image Analysis (ECG Buddy)

**Figure 2.**
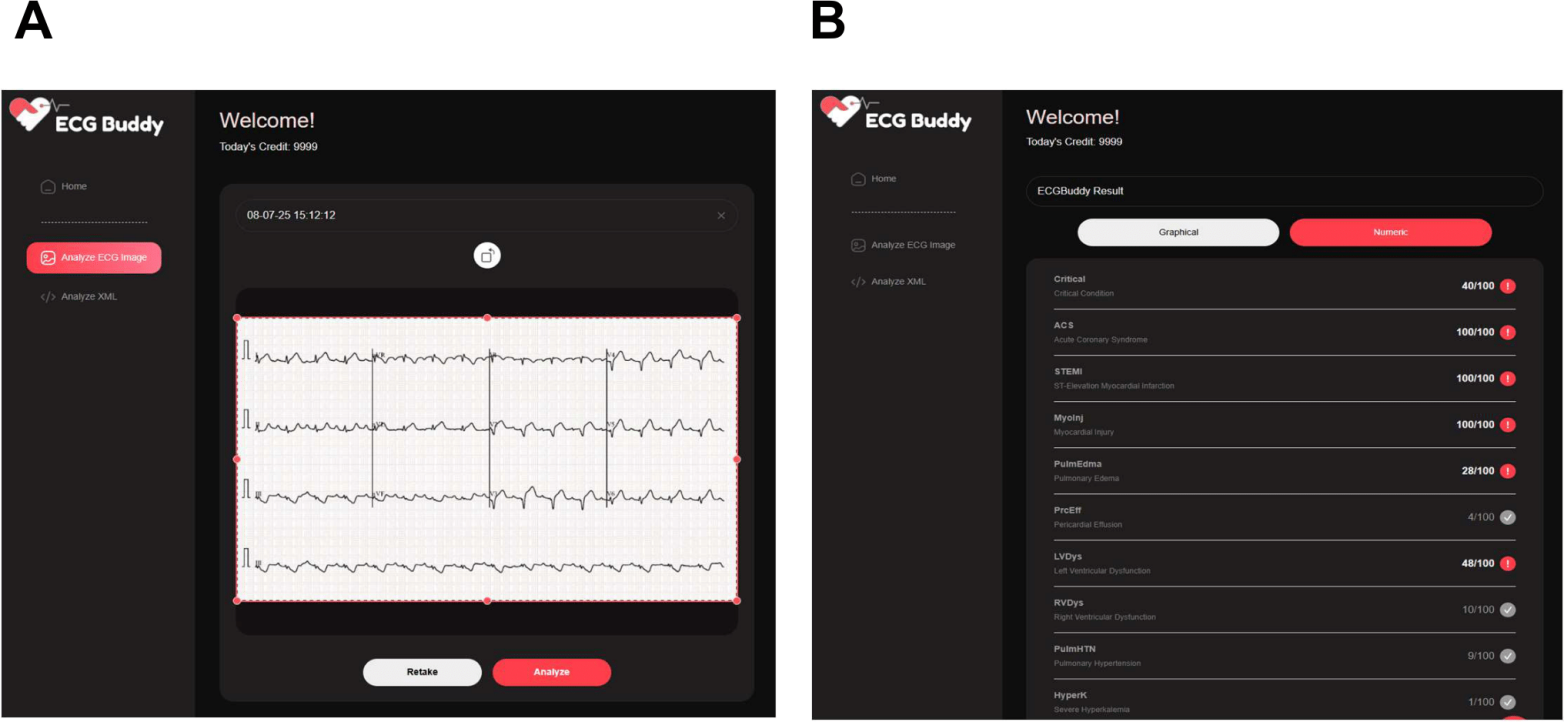
The operating screen of ECG Buddy for Microsoft Windows. (A) ECG input image and (B) ECG image analysis result.

ECG Buddy is a deep learning–based ECG analysis platform designed for 12-lead ECG image interpretation. The software is available for both smartphones and Microsoft Windows–based desktop personal computers. In this study, ECG Buddy for Microsoft Windows [21] was used to perform bulk analysis of ECG data (Figure 2). It is approved by the Korean Ministry of Food and Drug Safety and freely available for download in Korean app stores and can analyze 12-lead ECGs by taking pictures of ECG outputs to produce 10 digital biomarkers. The software automatically detects the ECG image displayed on the desktop and provides the analysis results within 10‐15 seconds. Figure 2A shows the operating screen of ECG Buddy for Microsoft Windows, while Figure 2B shows the ECG image analysis output. ECG Buddy generates 10 digital biomarkers that assess a range of cardiac conditions, including STEMI, acute coronary syndrome (ACS), myocardial injury (MyoInj), critical condition, pulmonary edema, pericardial effusion, left ventricular dysfunction, RV dysfunction, pulmonary hypertension, and severe hyperkalemia. This study analyzed only the STEMI, ACS, and MyoInj biomarkers owing to their direct relevance to MI classification.

### Statistical Analysis

Model performance was evaluated using accuracy, sensitivity, specificity, positive predictive value (PPV), and negative predictive value (NPV). The Youden index was used to determine optimal classification thresholds. In addition, model performance was evaluated using the area under the receiver operating characteristic curve (AUROC), and the AUC values were compared using the DeLong method, with statistical significance set at *P*<.05. All analyses were conducted using R software version 4.1.0 (RStudio) [22], with ChatGPT API responses obtained using Python (Python Software Foundation).

### Ethical Considerations

The study design was approved by the Institutional Review Board of Seoul National University Bundang Hospital (IRBX-2504-966-902). Given the public availability of the dataset, the Institutional Review Board of Seoul National University Bundang Hospital granted a waiver for the requirement of informed consent.

## Results

### Performance of ChatGPT and ECG Buddy

**Figure 3.**
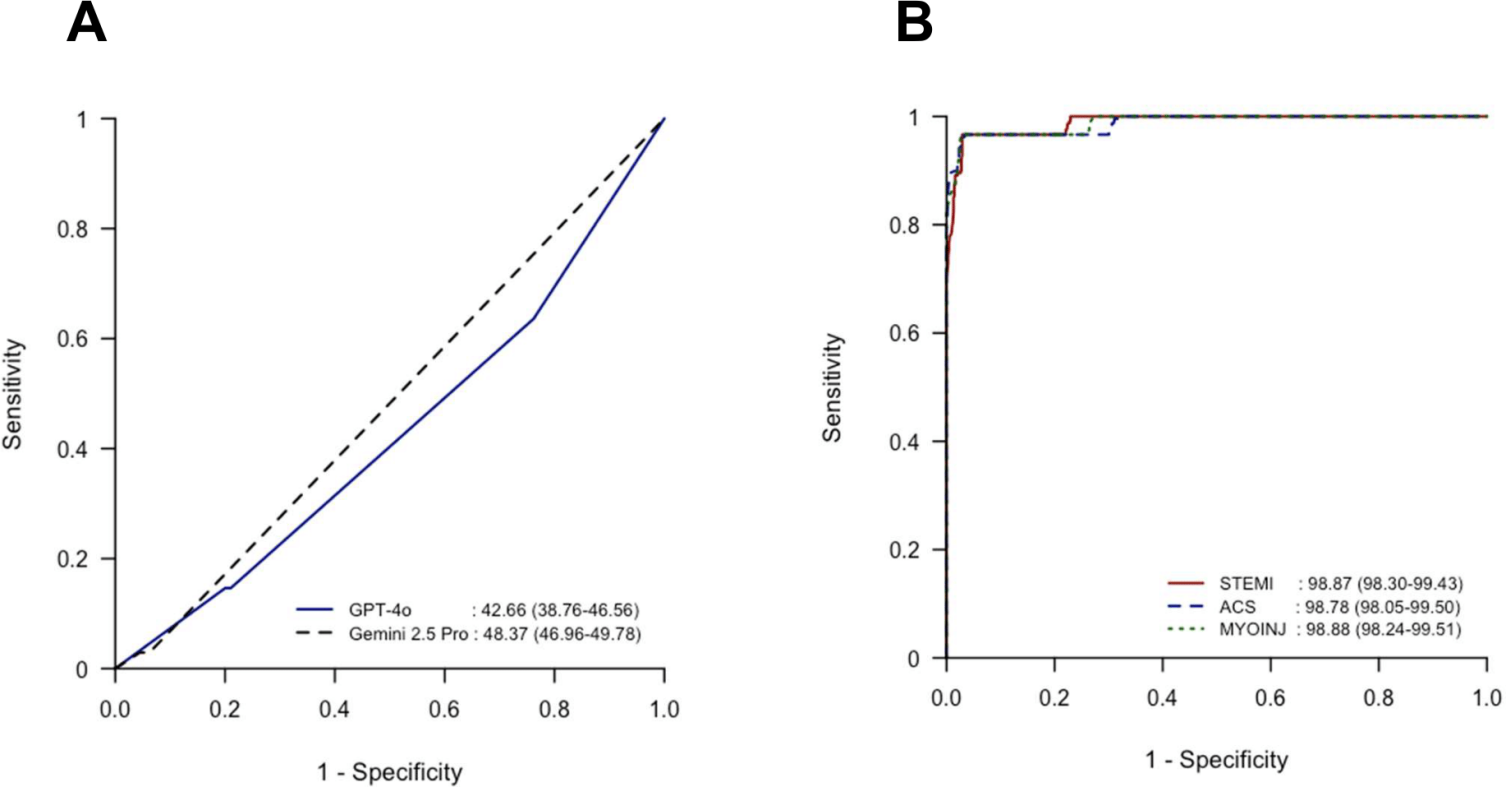
Diagnostic performance of large language models and ECG Buddy. (A) ChatGPT-4o and Gemini 2.5 Pro and (B) ECG Buddy. ACS: acute coronary syndrome; MYOINJ: myocardial injury; STEMI: ST-elevation myocardial infarction.

In total, 928 ECG recordings (239/928, 25.8% MI-positive cases) were analyzed, and all were successfully processed by both AI models. ChatGPT demonstrated limited discriminative ability in MI detection, achieving an AUC of 57.34% (95% CI 53.44‐61.24). Using the Youden index, the optimal cutoff was determined as the category “definite.” At this cutoff, the model’s sensitivity, specificity, PPV, and NPV were 36.40% (95% CI 30.30‐42.85), 76.20% (95% CI 72.84‐79.33), 34.66% (95% CI 28.79‐40.90), and 77.55% (95% CI 74.21‐80.64), respectively (Figure 3A and Table 1). Gemini 2.5 Pro showed even weaker overall discrimination, with an AUC of 51.63% (95% CI 50.22‐53.04). Applying the same Youden index procedure, the optimal cutoff corresponded to the “definite” category. At this threshold, sensitivity rose to 97.07% (95% CI 94.06‐98.81) but at the expense of specificity, which fell to 6.24% (95% CI 4.55‐8.31); the resulting PPV and NPV were 26.42% (95% CI 23.53‐29.47) and 86.00% (95% CI 73.26‐94.18), respectively (Figure 3A and Table 1).

The dedicated ECG AI software ECG Buddy exhibited highly accurate MI classification across the STEMI, ACS, and MyoInj markers. The AUC for detecting MI-positive cases for the STEMI biomarker was 98.87% (95% CI 98.30‐99.43), for the ACS biomarker was 98.78% (95% CI 98.05‐99.50), and for the MyoInj biomarker was 98.88% (95% CI 98.24‐99.51). Using the STEMI biomarker, ECG Buddy achieved the best accuracy of 96.98% (95% CI 95.67‐97.99), with a sensitivity of 96.65% (95% CI 93.51‐98.54), specificity of 97.10% (95% CI 95.55‐98.22), and *F*_1_-score of 94.27% (95% CI 91.86‐96.28). DeLong test confirmed that ChatGPT (AUC 53.63%) performed significantly worse than ECG Buddy across all biomarkers (all *P*<.001; Figure 3B and Table 1).

**Table 1.** Diagnostic performance of ChatGPT-4o, Gemini 2.5 Pro, and ECG Buddy. Data are expressed as values and 95% CI.

Metrics	ChatGPT-4o	Gemini 2.5 Pro	ECG Buddy		
			ST-elevation myocardial infarction	Acute coronary syndrome	Myocardial injury
Sensitivity (95% CI)	36.40 (30.30‐42.85)	97.07 (94.06‐98.81)	96.65 (93.50‐98.54)	96.65 (93.51‐98.54)	96.65 (93.51‐98.54)
Specificity (95% CI)	76.20 (72.84‐79.33)	6.24 (4.55‐8.31)	97.10 (95.55‐98.22)	96.66 (95.03‐97.87)	97.24 (95.73‐98.33)
PPV^a^ (95% CI)	34.66 (28.79‐40.90)	26.42 (23.53‐29.47)	92.03 (87.96‐95.07)	90.94 (86.72‐94.17)	92.40 (88.39‐95.36)
NPV^b^ (95% CI)	77.55 (74.21‐80.64)	86.00 (73.26‐94.18)	98.82 (97.68‐99.49)	98.81 (97.67‐99.49)	98.82 (97.69‐99.49)
AUROC^c^ (95% CI)	57.34 (53.44‐61.24)	51.63 (50.22‐53.04)	98.87 (98.30‐99.43)	98.78 (98.05‐99.50)	98.88 (98.24‐99.51)
Accuracy (95% CI)	65.95 (62.80‐69.00)	29.63 (26.71‐32.69)	96.98 (95.67‐97.99)	96.66 (95.29‐97.72)	97.09 (95.79‐98.07)

a PPV: positive predictive value.

b NPV: negative predictive value.

c AUROC: area under the receiver operating curve.

### Qualitative Assessment of Diagnostic Explanations of ChatGPT and Gemini

Two board-certified clinicians independently reviewed every explanation generated by the 2 LLMs. For GPT-4o, reviewer 1 judged 5% (2/40) explanations fully correct, 40% (16/40) partially correct, and 55% (22/40) completely incorrect. Reviewer 2 judged 5% (2/40) fully correct, 37.5% (15/40) partially correct, and 57.5% (23/40) completely incorrect. The reviewers concurred in 87.5% of GPT-4o cases (35/40; weighted κ=0.76). After consensus, GPT-4o explanations were fully correct in 5% (2/40), partially correct in 37.5% (15/40), and completely incorrect in 57.5% (23/40; Table 2).

For Gemini 2.5 Pro, the 2 clinicians showed high interrater agreement (91.9%, 34/37; weighted κ ≈ 0.67) while following the identical review procedure. Consensus ratings were 32.4% (12/37) fully correct, 13.5% (5/37) partially correct, and 54.1% (20/37) completely incorrect. Although Gemini produced a higher proportion of fully correct statements than GPT-4o, more than half of its explanations remained completely inaccurate, underscoring the need for expert oversight. The detailed per-rater counts are provided in Multimedia Appendix 1.

**Table 2.** Consensus qualitative assessment of large language model diagnostic explanations. Interrater agreement before consensus: κ=0.76 (GPT-4o) and 0.67 (Gemini).

	Correct, n (%)	Partially correct, n (%)	Completely incorrect, n (%)
GPT-4o (n=40)	2 (5)	15 (37.5)	23 (57.5)
Gemini 2.5 Pro (n=37)	12 (32.4)	5 (13.5)	20 (54.1)

## Discussion

### Principal Results

This study directly compared ChatGPT and Gemini, general-purpose multimodal LLMs, with ECG Buddy, a specialized deep-learning tool for ECG analysis. While ECG Buddy achieved high accuracy in detecting MI from ECG images, ChatGPT’s performance was significantly inferior. Gemini 2.5 Pro, the latest vision-language model from Google, showed even lower overall accuracy than GPT-4o, reinforcing the conclusion that current general-purpose LLMs remain unsuitable for primary ECG interpretation.

The considerable performance gap between the dedicated ECG Buddy and LLMs underscores a difference in their architecture and training methodologies. LLMs are primarily optimized for textual understanding and general visual recognition tasks and lack the specific training necessary for detailed ECG waveform interpretation. As a result, it may generate contextually plausible yet inaccurate responses, which could lead to potentially dangerous diagnostic errors if relied upon in clinical settings. Moreover, the performance of LLMs is highly sensitive to prompt design and the specific model version used, resulting in inconsistent outcomes. Clinical studies have reported that while LLMs perform moderately well in common clinical cases, they deviate significantly from evaluations in critical scenarios [10-12]. Conversely, ECG Buddy is optimized through targeted domain-specific training, resulting in superior performance and reliability compared to LLMs.

The findings of this study indicate that current general-purpose multimodal LLM architectures may primarily rely on textual annotations or explicit labels rather than on directly analyzing waveform patterns. Due to these structural limitations, current LLMs cannot be considered reliable as primary diagnostic tools for detecting STEMI. Accordingly, LLMs should be confined to a supplementary decision-support role, where they can supply guideline-based contextual information, augment the interpretations generated by specialized tools such as ECG Buddy, and propose clinically appropriate follow-up options. Although recent advances in LLMs have broadened the applications of medical AI, domain-specific models remain indispensable, as the inherent limitations of general-purpose LLMs still compromise clinical utility and reproducibility.

### Limitations

This study has some limitations. First, the study was conducted retrospectively using a publicly available ECG dataset, which lacked detailed clinical context or patient demographic information. The absence of comprehensive clinical data may limit the generalizability of these findings to diverse patient populations or different clinical settings. In addition, the dataset comprised only deidentified ECG images lacking infarct-territory labels and lab results, location-specific and STEMI or NSTEMI analyses were not possible. Second, the qualitative assessments were performed independently by 2 board-certified clinicians—1 emergency physician and 1 cardiologist. While providing useful insight, interpretations by multiple clinicians across various specialties might yield different assessments of diagnostic appropriateness or accuracy. To address this limitation and confirm its clinical utility, we plan to initiate prospective validation studies that will evaluate ECG Buddy’s diagnostic accuracy and workflow integration in emergency department settings across hospitals.

### Comparison With Prior Work

Our findings are consistent with those of prior research, highlighting that ECG-specialized AI models regularly outperform general-purpose models. Previous studies have demonstrated that deep learning models trained extensively on ECG-specific datasets accurately detect subtle waveform changes indicative of asymptomatic ventricular dysfunction and cirrhosis [23,24]. Similar to these specialized ECG models, ECG Buddy undergoes targeted optimization tailored specifically to ECG images, ensuring consistent predictive performance and stable error margins essential for clinical reliability. Notably, users only need to provide an ECG image or screenshot; both the smartphone and desktop versions feature an intuitive interface that requires no extra training or expertise. In addition to MI, ECG Buddy demonstrates robust diagnostic capabilities across diverse cardiac conditions, including STEMI, hyperkalemia, and RV dysfunction, validating its efficacy in various clinical settings [15-18]. Moreover, it has outperformed human experts in diagnosing STEMI and hyperkalemia.

### Conclusions

To our knowledge, this study provides the first direct comparative assessment between ChatGPT, Gemini, and ECG Buddy for detecting MI from ECG images. Our findings reveal that, despite the accelerating use of LLM-based AI, current LLMs do not meet the clinical performance and accuracy requirements for ECG interpretation. Herein, the ability of a general-purpose multimodal LLM (ChatGPT and Gemini) to detect ECG abnormalities fell short of that achieved by board-certified emergency physicians, rendering it insufficient for use in interpreting critical ECG readings in clinical practice. In contrast, the specialized ECG AI tool (ECG Buddy), which has been trained and validated, achieved high diagnostic accuracy and reproducibility, suggesting its utility in clinical settings. These results, consistent with those of several other studies, underscore the superiority of medical domain-specific AI programs over general-purpose LLMs for ECG analysis and interpretation and emphasize the importance of specialized models in the field of medical AI development.

## Supplementary material

10.2196/75910 Multimedia Appendix 1 Qualitative evaluation of large language model diagnostic explanations.

## Abbreviations

ACS: acute coronary syndrome
AI: artificial intelligence
API: application programming interface
AUC: area under the curve
ECG: electrocardiogram
LLM: large language model
MI: myocardial infarction
MyoInj: myocardial injury
NPV: negative predictive value
NSTEMI: non–ST-elevation myocardial infarction
PPV: positive predictive value
RV: right ventricular
STEMI: ST-elevation myocardial infarction
