# Supplementary material for: Comparative Diagnostic Performance of a Multimodal Large Language Model Versus a Dedicated Electrocardiogram AI in Detecting Myocardial Infarction From Electrocardiogram Images: Comparative Study
Source: JMIR AI. 2025 Sep 17;4:e75910. doi: 10.2196/75910 (PMC12443349; doi:10.2196/75910)
Supplement: Multimedia Appendix 1 [file ai-v4-e75910-s001.docx]

**Table S1.** Per-reviewer qualitative assessment of LLM diagnostic explanations

|  | Rater | correct | Partially correct | Completely incorrect |
| --- | --- | --- | --- | --- |
| GPT-4o | Reviewer 1 | 2/40 | 16/40 | 22/40 |
|  | Reviewer 2 | 2/40 | 15/40 | 23/40 |
|  | Consensus | 2/40 | 15/40 | 23/40 |
| Gemini 2.5 pro | Reviewer 1 | 12/37 | 4/37 | 21/37 |
|  | Reviewer 2 | 12/37 | 5/37 | 20/37 |
|  | Consensus | 12/37 | 5/37 | 20/37 |
